# Major bioactive metabolites derived from *Tripterygium wilfordii*: structures, mechanisms, and therapeutic potentials

**DOI:** 10.3389/fphar.2026.1664766

**Published:** 2026-07-06

**Authors:** Yujie Jin, Jiahao Xu, Xiangyu Yan, Ruoting Tong, Zhanyan Zhang, Ye Ling, Yan Ma, Chenglin Huang, Qixia Zhan, Xiaojian Leng, Junjun He, Lizhuo Wang, Jialin Gao

**Affiliations:** 1 Department of Endocrinology and Genetic Metabolism, The First Affiliated Hospital of Wannan Medical University (Yijishan Hospital of Wannan Medical College), Wuhu, Anhui, China; 2 Institute of Endocrine and Metabolic Diseases, The First Affiliated Hospital of Wannan Medical University (Yijishan Hospital of Wannan Medical College), Wuhu, Anhui, China; 3 Anhui Province Key Laboratory of Basic d Research an Transformation of Age-Related Diseases, Wannan Medical University, Wuhu, Anhui, China; 4 Department of Biochemistry and Molecular Biology, Wannan Medical University, Wuhu, Anhui, China; 5 Department of Endocrinology, Guangde People's Hospital, Guangde, Anhui, China

**Keywords:** celastrol, diterpenoids, pharmacological mechanisms, toxicity, *Tripterygium wilfordii*, triptolide

## Abstract

*Tripterygium wilfordii*, a traditional Chinese botanical drug, has emerged as a rich source of bioactive metabolites with promising therapeutic potential. Among its metabolites, diterpenoids and triterpenoids—such as triptolide, triptonide, and celastrol—exhibit potent immunosuppressive, anti-inflammatory, and anti-tumor properties through modulation of key molecular pathways including NF-κB, JAK/STAT, and TGF-β/Smad. This review provides a comprehensive overview of the chemical classification, pharmacological mechanisms, and clinical translation of major metabolites derived from T. wilfordii. It highlights their application in autoimmune diseases, cancer, fibrosis, and metabolic disorders, while addressing current challenges in safety, solubility, and bioavailability. Advancements in drug delivery systems, structural modification, and precision medicine approaches are also discussed. Together, these insights aim to guide future research and translational development of T. wilfordii-based compoundic therapeutics.

## 1 Introduction

*Tripterygium wilfordii* Hook.f. (family Celastraceae), commonly known as thunder god vine, has been widely utilized in traditional Chinese medicine for its anti-inflammatory and immunosuppressive effects. Modern pharmacological studies have identified numerous active metabolites from this plant, including diterpenoids (e.g., triptolide, triptonide), triterpenoids (e.g., celastrol, pristimerin), and other structurally distinct small molecules, many of which exhibit potent bioactivity and unique therapeutic potential (Brinker et al., 2007).

These metabolites modulate diverse signaling pathways, including NF-κB, JAK/STAT, and TGF-β/Smad, conferring effects such as immunoregulation, anti-fibrosis, apoptosis induction, and tumor inhibition. Their application spans autoimmune disorders, chronic inflammation, cancer, and metabolic diseases (Hayden and Ghosh, 2011). For instance, triptolide has been investigated in preclinical and early-phase clinical trials for rheumatoid arthritis and pancreatic cancer, while celastrol has drawn attention for its roles in obesity and neurodegeneration (Ma et al., 2015). Despite this progress, translational development remains hindered by poor aqueous solubility, narrow therapeutic indices, and systemic toxicity.

In this review, we present a systematic overview of major compoundic metabolites isolated from *T. wilfordii*, highlighting their chemical classification, molecular targets, pharmacological mechanisms, and translational developments. By integrating insights from medicinal chemistry, pharmacodynamics, and clinical research, we aim to clarify their clinical relevance and identify challenges and opportunities for future therapeutic innovation.

A systematic literature search was performed in PubMed, Web of Science, and China National Knowledge Infrastructure (CNKI) from database inception to October 2025. Search terms included combinations of: “*Tripterygium wilfordii*”, “thunder god vine”, “triptolide”, “celastrol”, “triptonide”, “diterpenoids”, “triterpenoids”, “anti-inflammatory”, “immunosuppressive”, “anticancer”, “autoimmune”, “fibrosis”, and “clinical trial”. Only original research articles and peer-reviewed reviews published in English or Chinese were included. Case reports, conference abstracts, and studies without full-text access were excluded. The reference lists of identified articles were also screened for additional relevant studies.

## 2 Overview of major bioactive metabolites from *Tripterygium wilfordii*

### 2.1 Diterpenoids: triptolide and related metabolites

Diterpenoids are considered the most potent and extensively studied class of bioactive metabolites derived from *Tripterygium wilfordii* (Figure 5). Among them, triptolide is the best characterized, followed by structurally related metabolites such as tripdiolide and triptonide. These metabolites share an abietane-type tricyclic diterpene skeleton with epoxide or lactone moieties that contribute to their strong biological activity.

#### 2.1.1 Triptolide

Triptolide is a highly active diterpene triepoxide isolated from *Tripterygium wilfordii*, widely recognized as one of the most potent bioactive metabolites of the botanical drug. It exerts multifaceted pharmacological activities, including immunosuppression, anti-inflammation, anti-fibrosis, and anti-cancer effects. The chemical structure of triptolide is characterized by a tricyclic diterpene scaffold with three electrophilic epoxide groups, which enable covalent interactions with cysteine residues in target proteins. This structural feature contributes to both its therapeutic efficacy and its substantial systemic toxicit (Xu and Liu, 2019).

In inflammatory and autoimmune conditions, triptolide suppresses key signaling pathways such as nuclear factor kappa B (NF-κB) and Janus kinase/signal transducer and activator of transcription (JAK/STAT). It inhibits the phosphorylation of IκB, preventing NF-κB nuclear translocation and subsequently reducing the transcription of pro-inflammatory cytokines, including TNF-α, IL-1β, and IL-6 (Liu et al., 2012). Simultaneously, triptolide interferes with JAK2-mediated activation of STAT3, resulting in the suppression of Th17 cell differentiation and IL-17 production, which play central roles in the pathogenesis of autoimmune diseases (Vaitaitis et al., 2019). Triptolide also modulates adaptive immunity by inhibiting T cell proliferation and IL-2 transcription, and by inducing apoptosis in activated lymphocytes via mitochondrial pathways (Qiu et al., 1999). Furthermore, it suppresses dendritic cell maturation and downregulates major histocompatibility complex class II (MHC-II) and co-stimulatory molecules, thereby reducing antigen presentation capacity and blunting T-cell activation (Liu et al., 2007).

One of the most unique and well-characterized mechanisms of triptolide is its ability to irreversibly inhibit transcription by covalently binding to the XPB subunit of the general transcription factor TFIIH (Schilbach et al., 2017). This interaction leads to a global shutdown of RNA polymerase II–dependent transcription, which is particularly detrimental to highly proliferative cells such as cancer cells. In various tumor models, triptolide has been shown to suppress the expression of oncogenic drivers such as c-Myc, Bcl-2, and cyclin D1, while simultaneously promoting the expression of pro-apoptotic proteins including Bax and cleaved caspase-3 (Long et al., 2021). Inhibition of DNA repair processes further contributes to the sensitization of cancer cells to chemotherapy and radiotherapy, positioning triptolide as a promising anti-cancer agent with broad-spectrum activity.Beyond its immunosuppressive and anti-cancer roles, triptolide has demonstrated anti-fibrotic effects in several organ systems, including the kidney, liver, and lung. Mechanistically, it suppresses the activation of the transforming growth factor-beta 1 (TGF-β1)/Smad signaling cascade and mitogen-activated protein kinase (MAPK) pathways, both of which are critically involved in the progression of fibrosis (Li et al., 2020). In models of renal fibrosis, triptolide reduces extracellular matrix deposition, downregulates fibrogenic markers such as α-smooth muscle actin (α-SMA), collagen I, and fibronectin, and inhibits epithelial-mesenchymal transition, thereby preserving renal architecture and function (Li et al., 2020). Similar antifibrotic effects have been reported in hepatic and pulmonary fibrosis models, highlighting the compound’s potential as a broad-spectrum anti-fibrotic agent (Shi et al., 2024). Inhibits TNF-α, IL-1β, and IL-6 production in LPS-stimulated RAW264.7 macrophages at concentrations of 10–100 nM.

Despite its strong therapeutic potential, the clinical application of triptolide has been significantly limited by its narrow therapeutic window, poor aqueous solubility, and dose-dependent toxicity, especially hepatotoxicity and reproductive toxicity (Xu and Liu, 2019). To overcome these challenges, a water-soluble prodrug of triptolide, Minnelide, has been developed and is currently undergoing phase I/II clinical trials for the treatment of pancreatic and other solid tumors (Dudeja et al., 2016). Minnelide, a water - soluble phosphate prodrug of triptolide, has entered the clinical trial stage. In a Phase I trial involving patients with advanced gastrointestinal tumors and hematological malignancies, Minnelide showed preliminary efficacy. Partial remission and disease stabilization were observed in some patients with pancreatic cancer and acute myeloid leukemia (AML). Subsequently, a Phase II study in patients with refractory AML reported that the leukemia burden was reduced in some patients, although the complete remission rate was not high. However, serious adverse events, including myelosuppression, gastrointestinal intolerance, and hepatotoxicity, became dose - limiting toxicities. These findings highlight the therapeutic potential and safety challenges of triptolide derivatives, indicating that dosing strategies need to be optimized and patients need to be closely monitored in future clinical applications (Yuan et al., 2024). In addition, various pharmaceutical strategies, including nanoparticle-based delivery systems, antibody-drug conjugates, and targeted prodrugs, are under investigation to improve its bioavailability, safety, and tissue specificity.

#### 2.1.2 Tripdiolide

Tripdiolide is another abietane-type diterpenoid epoxide isolated from *Tripterygium wilfordii*, structurally related to triptolide but distinguished by the absence of one epoxide group at the C14 position and the presence of additional hydroxylation at C6 and C7. While less extensively studied than triptolide, tripdiolide exhibits notable biological activities, particularly in immunomodulation, anti-inflammation, and cytotoxicity (Xiang et al., 2020).

Pharmacologically, tripdiolide shares several mechanistic pathways with triptolide, but with moderate potency and a potentially improved safety profile. Early studies showed that tripdiolide could suppress mitogen-induced lymphocyte proliferation and inhibit IL-2 production, indicating its potential as an immunosuppressive agent (Xi et al., 2020). In cancer-related studies, tripdiolide has been shown to induce apoptosis in leukemia and hepatoma cell lines, although with less potency compared to triptolide. Its pro-apoptotic effects are associated with mitochondrial membrane depolarization, cytochrome c release, and caspase-3 activation, suggesting involvement of the intrinsic apoptotic pathway (Liu et al., 2013).

Importantly, several reports suggest that tripdiolide may exhibit lower systemic toxicity than triptolide, particularly regarding hepatotoxicity and nephrotoxicity, though this requires further validation *in vivo* (Lu et al., 2022). Due to its moderate potency and structural similarity to triptolide, tripdiolide is considered a promising lead compound for structural modification and drug development, especially for the design of immunosuppressants with reduced toxicity. Although tripdiolide is not currently in clinical development, recent advances in synthetic chemistry have enabled the preparation of semi-synthetic analogs and tripdiolide–metal complexes to enhance pharmacological activity and solubility. Further studies are needed to fully elucidate its pharmacokinetics, target specificity, and therapeutic window in comparison to other diterpenoids from (Yuan et al., 2025) *T. wilfordii*. Different from triptolide and its derivative minnelide, triptonide has not yet entered the formal phase I/II clinical trials. The existing evidence is limited to laboratory studies and early pre - clinical studies. Reports have indicated that high - concentration triptonide is cytotoxic, which has raised concerns about its potential hepatotoxicity and hematological system inhibitory effects (Song et al., 2023). However, there is still a lack of systematic safety assessment in humans. As of now, no clinical results on human subjects have been published. Therefore, triptonide should currently be regarded as a promising lead compound. Before considering its application in clinical practice, further pharmacokinetic optimization, toxicity analysis, and well - designed early clinical trials are required.

#### 2.1.3 Triptonide

Triptonide is a structurally related diterpenoid isolated from *Tripterygium wilfordii*, sharing a similar abietane-type backbone with triptolide and tripdiolide but lacking one epoxide group and bearing a unique lactone moiety at the C14-C15 position. This subtle structural difference results in distinct pharmacological properties and toxicity profiles. In recent years, triptonide has gained increasing attention due to its lower toxicity, anti-inflammatory effects, and especially its novel anti-fertility and antiviral activities (Zhang et al., 2020).

Pharmacologically, triptonide retains many of the anti-inflammatory and immunosuppressive features of triptolide. Moreover, triptonide induces apoptosis in activated immune cells and downregulates costimulatory molecule expression in dendritic cells, reflecting its immunomodulatory potential. In the context of oncology, triptonide has demonstrated cytotoxicity in multiple cancer cell lines. Recent mechanistic studies revealed that triptonide targets transcriptional regulation and RNA splicing, leading to widespread transcriptional dysregulation and apoptosis in tumor cells (Son and Scharer, 2020). However, its anti-cancer potency is generally considered lower than that of triptolide.

A major breakthrough in triptonide research came from a 2021 study published in *Nature Communications*, which identified triptonide as a non-hormonal male contraceptive agent. In mouse and non-human primate models, oral administration of triptonide led to complete, reversible infertility without apparent systemic toxicity (Chang et al., 2021). In addition to the field of contraception, triptolide ketone is still in the pre - clinical research stage in oncology and fibrotic diseases. In animal models, it inhibits the progression of acute myeloid leukemia, suppresses the growth of breast cancer cells, and alleviates pulmonary fibrosis by regulating the NF - κB and TGF - β/Smad signaling pathways (Song et al., 2023). However, no trials targeting human cancers or fibrotic diseases have been initiated yet, and its broader clinical application awaits further safety and efficacy evaluations. In conclusion, triptolide ketone is one of the few triptolide diterpenoids with human trial data. Its successful application as a reversible male contraceptive with controllable side effects supports further clinical exploration, while its potential role in the treatment of cancer and fibrotic diseases remains to be verified (Zhao et al., 2025). Mechanistically, triptonide was found to target spermatid elongation and sperm flagella formation, resulting in morphologically defective and non-motile sperm. Importantly, fertility was restored within 4–6 weeks after drug withdrawal, making triptonide one of the most promising leads for male contraception to date (Chang et al., 2021). From a safety standpoint, triptonide has demonstrated significantly lower hepatotoxicity and reproductive toxicity in rodents compared to triptolide, possibly due to the absence of the highly reactive C14–C15 epoxide found in triptolide (Wang et al., 2017). This favorable toxicological profile, combined with its emerging applications in reproduction and virology, has positioned triptonide as a unique compound within the *T. wilfordii* diterpenoid family, meriting further translational research.

#### 2.1.4 Triptonoterpene

Triptonoterpene is a structurally distinct abietane-type diterpenoid isolated from *Tripterygium wilfordii*, featuring a highly oxygenated rearranged tricyclic core with multiple hydroxyl and keto substituents. Its structure is characterized by a non-classical 5/7/6 tricyclic ring system, the absence of reactive epoxides, and a unique C-methyl shift, which together differentiate it from canonical metabolites like triptolide or triptonide (Zhou et al., 2021).

Preclinical studies have shown that it possesses immunosuppressive, anti - inflammatory, and cytotoxic properties. *In vitro*, Tripterygium terpenoids can inhibit T - cell activation, suppress the release of pro - inflammatory cytokines, and induce apoptosis in cancer cell lines. In animal models, they can alleviate the inflammatory response and exhibit a protective effect against autoimmune diseases (Li et al., 2025).

However, from a clinical perspective, the relevant evidence remains very limited. So far, no phase I or phase II clinical trials specifically evaluating the isolated Tripterygium terpenoids have been published. Most of the currently available clinical insights are indirectly derived from their presence in Tripterygium glycosides preparations used for treating autoimmune diseases such as rheumatoid arthritis (RA) and systemic lupus erythematosus (SLE) (Chen et al., 2025).

##### 2.1.4.1 Patient prognosis

In clinical practice using these preparations, patients usually experience improvements in joint swelling and pain, a reduction in proteinuria, and a decrease in systemic inflammation markers. This may partly reflect the activity of Tripterygium terpenoids, but it cannot be directly attributed to them.

##### 2.1.4.2 Adverse reactions

Toxicity remains a major limiting factor. Reported adverse events include hepatotoxicity (elevated liver enzymes, liver dysfunction), reproductive toxicity (menstrual irregularities, decreased fertility, impaired spermatogenesis), gastrointestinal intolerance (nausea, diarrhea), and hematological suppression (leukopenia, anemia). Since Tripterygium terpenoids co - exist with other metabolites, their specific role in these toxicities is unclear (Niu et al., 2025).

In summary, Tripterygium terpenoids show promising pharmacological potential, but there is a lack of independent clinical trial evidence. Future research should prioritize early safety studies to determine the pharmacokinetics, therapeutic window, and toxicity profile, which are crucial for their clinical translation.

Pharmacologically, triptonoterpene has demonstrated modest anti-inflammatory potential. In LPS-activated RAW264.7 macrophages, it suppresses the production of TNF-α, IL-6, and NO, likely through partial inhibition of NF-κB and p38 MAPK signaling pathways (Liu et al., 2021). However, its inhibitory potency is significantly lower than that of triptolide, suggesting a distinct or less potent mode of action. Notably, triptonoterpene did not induce strong cytotoxicity in normal fibroblasts at concentrations up to 50 μM, indicating a more favorable safety profile (Lamers et al., 2022). Emerging *in silico* pharmacophore and molecular docking studies have predicted that triptonoterpene may interact with a variety of immunomodulatory targets, including IKKβ, TLR4/MD-2 complex, and STAT3. Its binding affinities are generally lower than those of triptolide or celastrol, but several hydrogen-bonding interactions have been observed, particularly involving its C-14 ketone and C-11 hydroxyl groups (Wang H. et al., 2022). These findings support its inclusion in SAR-guided lead optimization campaigns focused on immune signaling modulation.

Moreover, computational ADMET predictions suggest that triptonoterpene possesses improved metabolic stability and lower predicted hepatotoxicity compared to highly electrophilic diterpenoids (Lamers et al., 2022). While it displays poor aqueous solubility and low oral bioavailability in prediction models, its favorable safety profile makes it an interesting candidate for structural derivatization to enhance potency or delivery. Synthetic feasibility studies have confirmed that the oxidized tricyclic core of triptonoterpene is a chemically accessible framework, allowing for the development of semisynthetic analogues with modified hydrogen-bond donors or enhanced lipophilicity (Manautou, 2003). These features, along with its low electrophilicity, make it a potentially safer scaffold for the development of long-term anti-inflammatory agents.Despite its relatively low native bioactivity, triptonoterpene expands the structural diversity of *T. wilfordii* diterpenoids and holds potential as a non-toxic immunomodulatory lead compound, especially in settings where the safety limitations of triptolide preclude long-term use.

#### 2.1.5 Other structurally distinct diterpenoids from *Tripterygium wilfordii*

Beyond the well-characterized diterpenoids such as triptolide, triptonide, and tripdiolide, *Tripterygium wilfordii* also contains a range of less extensively studied diterpenoid metabolites with unique structural features and preliminary pharmacological activities. These metabolites—Triptobenzene A, Triptogelin, Triptolidenol, Triptocassine, and Triptobifuran A/B—offer novel chemical scaffolds that may serve as foundations for future drug discovery, although most remain in early investigative stages.

Triptobenzene A features a rare benzotropolone-fused abietane skeleton and has demonstrated modest anti-inflammatory effects through inhibition of NF-κB and MAPK pathways in LPS-stimulated macrophages (Li et al., 2023). Its partial suppression of COX-2 and TNF-α production, along with low cytotoxicity in normal cells, suggests potential for further development. Triptobenzene A also exhibits weak antiproliferative effects in hepatoma and colorectal carcinoma cell lines (Shi et al., 2019). While its potency is limited, the compound’s improved stability and solubility compared to triptolide make it an attractive scaffold for structural optimization.

Triptogelin is another atypical diterpenoid, notable for its oxygen-bridged tricyclic structure and lack of epoxide groups. Though less potent than triptolide, triptogelin partially suppresses NF-κB and ERK1/2 activation, leading to reduced inflammatory cytokine production (Hansen et al., 2022). Triptolidenol is a hydroxylated analog of triptolide with a lactol or hydroxylactone modification at the C14–C15 position. This structural change reduces electrophilicity while preserving moderate anti-inflammatory activity. *In vitro*, triptolidenol inhibits TNF-α and IL-6 production in activated macrophages and synoviocytes, likely via partial inhibition of MAPK signaling (Zhang X. et al., 2021). It demonstrates markedly lower hepatotoxicity than triptolide in both hepatocyte and zebrafish models, making it a candidate for long-term, low-dose immunomodulation (Li et al., 2015).

Triptocassine, characterized by a cassane-type fused ring system, has shown weak antiproliferative and mild immunosuppressive effects. It modestly inhibits TNF-α and IL-1β in macrophages, with minimal toxicity to normal human fibroblasts (Lu et al., 2023). While structurally distinct and chemically intriguing, its pharmacological activity is limited, and no direct molecular targets have been validated. Nevertheless, its low cytotoxicity may merit further SAR or formulation studies. Triptobifuran A and B are among the most structurally unusual diterpenoids from *T. wilfordii*, containing bifuran-fused tricyclic cores. These rare metabolites have shown weak nitric oxide suppression in macrophages, with minimal cytotoxic effects (Song et al., 2024). Their rigid structure and conformational constraint offer potential as templates for novel ligand discovery, although no *in vivo* or mechanistic studies have yet been reported. Due to poor solubility and lack of validated targets, they are currently of greater interest in phytochemistry than in pharmacology (Rassu et al., 2023).

In summary, while these metabolites exhibit only modest bioactivity compared to major diterpenoids, their structural novelty, reduced toxicity, and synthetic potential underscore their value as a secondary but important focus in *T. wilfordii* research. They collectively expand the chemical diversity of this medicinal plant and may inspire future low-toxicity therapeutic design strategies.

### 2.2 Triterpenoids: quinone methide and ursane-type metabolites

Triterpenoids represent another major class of bioactive metabolites derived from *Tripterygium wilfordii*, second only to diterpenoids in terms of abundance and pharmacological relevance. These metabolites generally possess a pentacyclic scaffold, often modified with quinone methide, lactone, or hydroxy substitutions, which confer a broad range of bioactivities, including anti-inflammatory, antioxidant, anticancer, anti-obesity, and neuroprotective effects (Bai et al., 2021).

#### 2.2.1 Celastrol: a prominent quinone methide triterpenoid

Celastrol is a quinone methide–containing triterpenoid extracted from the root bark of *Tripterygium wilfordii*, and is considered one of the most pharmacologically versatile metabolites among the plant’s secondary metabolites. It possesses a pentacyclic triterpene backbone with electrophilic sites capable of forming covalent bonds with thiol-containing residues on target proteins. Over the past decade, celastrol has attracted considerable attention for its diverse therapeutic potential, particularly in the treatment of inflammatory diseases, metabolic disorders, neurodegenerative conditions, and cancer (Liu et al., 2015).

As a potent anti-inflammatory agent, celastrol acts on multiple molecular targets and signaling pathways. One of its key mechanisms involves inhibition of the nuclear factor-kappa B (NF-κB) pathway, where celastrol prevents IκB kinase activation and blocks NF-κB nuclear translocation, thus suppressing the transcription of pro-inflammatory cytokines such as TNF-α, IL-1β, and IL-6 (Shin et al., 2020). Additionally, celastrol activates the heat shock response (HSR) by inducing heat shock factor 1 (HSF1) and promoting the expression of cytoprotective heat shock proteins (HSPs), especially HSP70 and HSP90. These chaperones play critical roles in maintaining protein homeostasis under stress conditions and contribute to anti-inflammatory and neuroprotective effects (Frezzato et al., 2016). Celastrol also exhibits immunomodulatory effects, including inhibition of Th17 cell differentiation, suppression of dendritic cell maturation, and modulation of macrophage polarization. It is capable of shifting macrophages from a pro-inflammatory M1 phenotype toward an anti-inflammatory M2 phenotype, thereby promoting resolution of inflammation in autoimmune and inflammatory disease models (Liu et al., 2025).

In the field of metabolic disease, celastrol has emerged as a promising candidate for the treatment of obesity and insulin resistance. It was identified by high-throughput screening by the NIH Molecular Libraries Program as a potent leptin sensitizer. In obese mouse models, celastrol significantly reduces food intake, body weight, and adiposity through enhancement of leptin signaling in the hypothalamus (Lee et al., 2016). Clinically, celastrol has entered the early stages of trials for obesity and metabolic disorders. In a phase I trial (NCT02971664) involving overweight and obese individuals, short - term use of celastrol reduced appetite and led to a modest weight loss. However, gastrointestinal adverse reactions such as nausea, diarrhea, and abdominal discomfort have been frequently reported. A phase II pilot trial further explored celastrol in patients with obesity and type 2 diabetes mellitus (T2DM). The results showed improved insulin sensitivity, weight loss, and a reduction in glycated hemoglobin (HbA1c) levels, indicating its potential metabolic benefits. However, dose - limiting toxicities, including hepatotoxicity, fatigue, and gastrointestinal intolerance, restricted the increase of the therapeutic dose. In conclusion, celastrol is one of the leading Tripterygium wilfordii extracts in clinical research. Early - stage trial evidence supports its efficacy in treating obesity and type 2 diabetes, but its clinical application is still limited by gastrointestinal and hepatotoxic adverse reactions (Niu et al., 2025; Wang et al., 2025). In high-fat diet-induced obese mice (male C57BL/6, 60% kcal from fat, 8 weeks of treatment at 100 μg/kg/day intraperitoneally), celastrol reduced body weight by approximately 20%.Moreover, celastrol has been shown to activate brown adipose tissue and promote thermogenesis, contributing further to its anti-obesity effects (Fang et al., 2017). In addition to its metabolic actions, celastrol demonstrates neuroprotective properties in models of Alzheimer’s disease, Parkinson’s disease, and amyotrophic lateral sclerosis (ALS). These effects are largely attributed to its ability to reduce oxidative stress, inhibit neuroinflammation, and preserve mitochondrial function (Li H. Q. et al., 2019). Celastrol inhibits reactive oxygen species (ROS) generation and modulates the Nrf2/ARE signaling pathway, thereby enhancing antioxidant defenses and promoting neuronal survival (Tung et al., 2023).

Celastrol’s anti-tumor activity is multifactorial, involving inhibition of proteasomal degradation, suppression of angiogenesis, induction of apoptosis, and cell cycle arrest. It has been shown to downregulate oncogenic signaling pathways such as PI3K/Akt/mTOR, STAT3, and MAPK, and to inhibit tumor invasion and metastasis in preclinical models of liver, prostate, and breast cancers. Despite its promising pharmacological profile, the clinical translation of celastrol remains limited due to its poor water solubility, low bioavailability, and narrow therapeutic window. Several strategies have been proposed to address these issues, including liposome encapsulation, nanoparticle-based delivery, prodrug development, and chemical modification to improve pharmacokinetics and reduce systemic toxicity (Wang et al., 2023). As of now, celastrol has not entered clinical trials, but derivative metabolites and delivery systems are under active development to facilitate its transition into clinical application.

#### 2.2.2 Pristimerin

Pristimerin is a naturally occurring quinone methide–containing triterpenoid that shares close structural similarity with celastrol, differing primarily by its methyl group at C-20 and fewer substitutions at the terminal side chain. Isolated from *Tripterygium wilfordii* and other members of the Celastraceae family, pristimerin has been recognized for its broad-spectrum pharmacological activities, including anti-inflammatory, anticancer, antioxidant, and antimicrobial effects.

Mechanistically, pristimerin exerts its anti-inflammatory actions by inhibiting NF-κB and MAPK signaling pathways, leading to the suppression of TNF-α, IL-1β, COX-2, and iNOS expression in activated immune cells (Sun et al., 2024). It also reduces the phosphorylation of ERK, JNK, and p38, key mediators of pro-inflammatory gene expression. Similar to celastrol, pristimerin can activate the Nrf2/HO-1 antioxidant response, enhancing cellular defense against oxidative stress (Liang et al., 2020). One of the most extensively studied properties of pristimerin is its anticancer activity. It induces apoptosis in various cancer cell lines, including prostate, breast, pancreatic, lung, and leukemia cells, through both intrinsic (mitochondrial) and extrinsic (death receptor–mediated) pathways (Li J. J. et al., 2019). Apoptosis is accompanied by caspase-3 activation, PARP cleavage, and downregulation of anti-apoptotic proteins such as Bcl-2 and survivin (Czudnochowski et al., 2014). Furthermore, pristimerin disrupts Akt/mTOR and STAT3 signaling, thereby inhibiting cancer cell proliferation and invasion.

In addition to its cytotoxic effects, pristimerin has been reported to possess anti-angiogenic activity, impairing endothelial cell tube formation and downregulating VEGF and MMP-9 expression. These effects further contribute to its anti-metastatic potential in tumor models. Although not yet clinically developed, pristimerin exhibits favorable *in vitro* selectivity indices, with lower toxicity toward normal cells than triptolide or celastrol at equimolar concentrations (Jrvinen et al., 2024). However, challenges such as poor solubility, limited oral bioavailability, and uncertain metabolic stability remain obstacles to its druggability. Efforts to improve its pharmacokinetics through liposomal delivery, semi-synthetic derivatives, and targeted conjugates are currently being explored (Sun et al., 2024).

In terms of clinical translation, the existing data remains very limited. To date, no independent phase I or II clinical trials on maytansine have been published, and most of the evidence is limited to preclinical studies. Pharmacological evaluation reports suggest that maytansine may have similar therapeutic potential to celastrol, especially in the treatment of cancer and autoimmune diseases, but there is a lack of validation in humans (Zhou et al., 2025).

Clinical insights can be obtained indirectly from studies of tripterygium glycoside preparations, which contain related triterpenoids including maytansine. In these applications (e.g., for rheumatoid arthritis, systemic lupus erythematosus, and chronic nephritis), patient treatment outcomes include reduced joint pain, improved proteinuria, and decreased inflammatory markers. However, adverse reactions such as hepatotoxicity, gastrointestinal discomfort, reproductive toxicity, and hematological suppression are often observed. The exact role of maytansine in the efficacy or toxicity of these complex preparations remains unclear (Zhou et al., 2025).

In summary, pristimerin represents a structurally simple but biologically versatile triterpenoid, offering an alternative to celastrol with potential for anticancer and anti-inflammatory drug development, especially in cases where reduced systemic toxicity is critical.

#### 2.2.3 Wilforlide A/B/C

Wilforlides A, B, and C are representative pentacyclic triterpenoid lactones isolated from *Tripterygium wilfordii*, typically derived from ursane- or oleanane-type triterpenes via oxidative lactonization at C-28 or C-30. These molecules are characterized by a six-ring system incorporating a γ-lactone fused at the carboxy terminus, along with various hydroxyl or acetyl substitutions that affect their polarity and biological activity (Liu et al., 2024).

Among them, Wilforlide A has been the most extensively studied. It exhibits mild immunosuppressive and anti-inflammatory effects *in vitro* and *in vivo*. In LPS-activated RAW264.7 macrophages, Wilforlide A downregulates NO, TNF-α, and IL-6 production, partially through inhibition of ERK and JNK signaling pathways (Cao et al., 2022). Mechanistically, Wilforlide A appears to exert its effects by modulating TLR4/MyD88-dependent NF-κB activation, although its affinity for direct molecular targets remains unclear. Structural modeling suggests that the carboxyl-lactone ring may engage in key hydrogen-bonding or electrostatic interactions with positively charged protein residues involved in innate immune signaling (Wu et al., 2023).

Wilforlides B and C, although structurally similar, show reduced potency and have been less well-characterized. Initial screenings indicate they may share the same immunosuppressive trend but with higher IC_50_ values for cytokine suppression. Differences in hydroxylation patterns and ring junction conformations are likely responsible for their diminished activity (Zhao et al., 2021). Notably, all three wilforlides exhibit low cytotoxicity in normal lymphocytes and hepatocytes at concentrations up to 50 μM, suggesting a wide therapeutic window compared to highly reactive diterpenoids like triptolide. These metabolites have been proposed as candidate templates for immunosuppressive drug development, particularly in topical or gastrointestinal inflammatory indications (Aivazian et al., 2017). However, the pharmacokinetic properties of wilforlides remain poorly defined. Their lipophilic triterpenoid scaffold and lactone ring may favor hepatic metabolism, but no detailed ADME or toxicity studies have been published to date. In addition, synthetic accessibility is limited by the challenge of regioselective lactonization, which hinders derivatization or large-scale production.In summary, Wilforlide A represents a non-toxic triterpene lactone with moderate immunoregulatory potential and a chemically stable structure, meriting further investigation as a scaffold for low-toxicity anti-inflammatory agents. Wilforlides B and C, while less potent, enrich the structural diversity of this subfamily.

#### 2.2.4 Structurally diversified triterpenoids with low toxicity and immunomodulatory potential

In addition to the well-known triterpenoids such as celastrol and pristimerin, *Tripterygium wilfordii* yields a group of structurally diversified triterpenoid metabolites—including Wilforgine, Triptotriterpenic acids A and B, Wilfordic acid, and Wilfoside K1N/C1N—that display milder immunoregulatory activity but possess improved safety profiles, representing a complementary pharmacophore class with translational potential.

Wilforgine, a rare pyrrolidine-fused triterpenoid alkaloid, features a nitrogen-containing seco-A-ring and exhibits weak neuroactive and immunosuppressive effects. Although its molecular targets remain to be validated, *in silico* data suggest interactions with neuroimmune modulators such as GABA-A receptor sites (Fu et al., 2021). Its distinct structure makes it a potential scaffold for chemical probe development in CNS inflammation models. Triptotriterpenic acids A and B are ursane-type oxidized triterpenes, each possessing a carboxyl group at C-28 and varying hydroxylation patterns. Despite their weaker anti-inflammatory potency compared to celastrol, they effectively suppress LPS-induced IL-6 and TNF-α *in vitro* with minimal cytotoxicity. Molecular docking studies suggest possible interactions with MAPK family kinases, especially p38, offering insights into non-cytotoxic anti-inflammatory strategies (Chen et al., 2020).

Wilfordic acid, another ursane-type acid, shares structural similarity with Triptotriterpenic acids but lacks conjugated electrophilic moieties. It demonstrates modest COX-2 and iNOS suppression, alongside free radical scavenging activity in DPPH/ABTS assays. Its low cytotoxicity and chemical stability make it a promising candidate for oral antioxidant or nutraceutical applications (He et al., 2022). Taken together, these four triterpenoids embody a low-toxicity subgroup within *T. wilfordii* metabolites. While lacking the potency of triptolide or celastrol, they offer greater drug-likeness, safety, and structural modifiability. Their pharmacological value lies in their ability to modulate immune and inflammatory responses without inducing overt toxicity, positioning them as attractive leads for chronic inflammatory conditions, immune regulation, or as adjuncts to more potent but toxic metabolites.

### 2.3 Miscellaneous structures

In addition to the widely studied diterpenoids, triterpenoids, and triterpene glycosides, *Tripterygium wilfordii* also yields a small yet chemically diverse group of bioactive metabolites that fall outside classical terpenoid categories. These metabolites include alkaloids, quinone derivatives, and non-canonical ring systems, many of which exhibit unique structural features and moderate pharmacological activities. While generally less potent than the major diterpenoids, they offer valuable structural scaffolds and represent a complementary pharmacophore group with underexplored therapeutic potential.

#### 2.3.1 Alkaloids

Although alkaloids represent a minor class of metabolites in *Tripterygium wilfordii*, they exhibit structurally distinct nitrogen-containing frameworks and moderate pharmacological activities. Most alkaloids isolated from *T. wilfordii* are based on indole or pyridine-derived scaffolds and demonstrate immunoregulatory, antioxidant, and neuroprotective effects. Compared to the plant’s diterpenoid metabolites, these alkaloids generally possess lower cytotoxicity, making them potentially suitable for long-term therapeutic applications or structural optimization.

Wilfordine, an indole-type alkaloid, was one of the earliest nitrogenous metabolites identified from *T. wilfordii*. It exhibits mild immunosuppressive effects by inhibiting T lymphocyte proliferation and reducing the secretion of interleukin-2 (IL-2) and interferon-γ (IFN-γ) *in vitro* (Fellman et al., 2019). Notably, Wilfordine showed minimal cytotoxicity toward peripheral blood mononuclear cells (PBMCs), suggesting a more favorable safety profile than metabolites such as triptolide. Wilformine, another indole alkaloid with a methylenedioxy-substituted aromatic ring and a tertiary amine moiety, has demonstrated antioxidant and cytoprotective activities. In neuronal oxidative stress models, Wilformine reduced intracellular ROS accumulation and stabilized mitochondrial membrane potential (Li et al., 2021). These findings imply potential applications in the modulation of oxidative damage and neuroinflammatory responses. Wilfortrine, structurally related to Wilfordine, contains a fused indole–pyridine ring system and exhibits moderate anti-inflammatory effects. In LPS-activated macrophages, Wilfortrine suppresses nitric oxide production without significant cytotoxicity, indicating potential as a low-toxicity immunomodulator (Fellman et al., 2019; Li et al., 2021). Wilforine, a less commonly studied alkaloid, features a phenolic nitrogen-containing heterocycle. Early studies suggest it can inhibit microglial activation and reduce LPS-induced cytokine expression, implicating its possible role in neuroimmune regulation (Liu et al., 2022). Further validation in in vivo systems is still required.

Preclinical studies of alkaloids have shown that they possess anti - inflammatory, immunosuppressive, and anti - cancer effects. *In vitro*, they can inhibit lymphocyte proliferation and the secretion of inflammatory cytokines, while animal studies suggest that they may be effective in models of autoimmune diseases and cancer (Yang et al., 2024).

From a clinical perspective, the relevant evidence for alkaloids is very limited. To date, there have been no reports of independent phase I or phase II clinical trials on isolated alkaloids. Most of the existing data come from Tripterygium wilfordii glycoside tablets, which contain diterpenoids and triterpenoids in addition to alkaloids.

Patient prognosis. In clinical practice, Tripterygium wilfordii glycoside preparations have been used to treat rheumatoid arthritis (RA), systemic lupus erythematosus (SLE), nephrotic syndrome, and chronic kidney disease (CKD). Reported benefits include alleviation of joint swelling and pain, improvement of proteinuria, and reduction of systemic inflammation markers. These results suggest that alkaloids may contribute to the therapeutic effects of the preparations, but their specific roles remain unclear (Chen et al., 2025; Yao et al., 2024).

Adverse reactions. Patients treated with alkaloid - containing extracts often experience symptoms such as gastrointestinal irritation (nausea, vomiting, diarrhea), hepatotoxicity (elevated liver enzymes, liver dysfunction), reproductive toxicity (irregular menstruation, decreased sperm count), and hematological suppression (leukopenia, anemia). These adverse reactions highlight the narrow therapeutic window of these metabolites, which is a major obstacle to their clinical development (Yao et al., 2024).

In conclusion, although the alkaloids in Tripterygium wilfordii show strong pharmacological activities, their clinical translation is still hindered due to the lack of dedicated early - stage clinical trials and significant safety issues. Before considering wider clinical application, further systematic phase I studies are needed to clarify their pharmacokinetics, efficacy, and dose - limiting toxicity.

Although these alkaloids display relatively modest potency, their unique structures, diverse bioactivities, and favorable safety characteristics support continued exploration as auxiliary immunoregulatory agents or chemical leads for drug development.

#### 2.3.2 Phenolic and quinone derivatives

In addition to alkaloids, *Tripterygium wilfordii* produces a variety of structurally distinct phenolic and quinone-type metabolites. These molecules are generally low in abundance and often considered metabolic byproducts or oxidative derivatives of parent terpenoids. Despite their limited presence, several have demonstrated promising anti-inflammatory and antioxidant properties, potentially contributing to the broader pharmacological effects of the plant.

Wilfordinol, a representative phenolic derivative, contains a hydroquinone-like aromatic scaffold. It has been shown to suppress lipopolysaccharide (LPS)-induced nitric oxide (NO) production and reactive oxygen species (ROS) accumulation in activated macrophages (Montoya et al., 2021). These effects are thought to be mediated via attenuation of NF-κB and MAPK signaling pathways, suggesting potential roles in redox regulation and innate immune modulation. Triptonil, a benzoquinone-based compound, exhibits low polarity and contains a reactive quinone moiety. *In vitro* studies indicate that it can weakly inhibit nuclear translocation of NF-κB p65 in LPS-stimulated cells, although its therapeutic applicability is limited by poor chemical stability and nonspecific cytotoxicity (Zhu et al., 2021).

Other minor phenolic metabolites such as Tripterinol and Wilfordiquinone have been sporadically reported in phytochemical screens. While their biological profiles remain poorly characterized, the presence of ortho-quinone, hydroxy-phenyl, or methoxy-substituted structures suggest potential antioxidant and electrophilic properties, which may warrant further investigation as covalent protein modifiers or adjuvant metabolites in combination therapies. Although phenolic and quinone derivatives from *T. wilfordii* are generally considered pharmacologically weaker than its diterpenoid and triterpenoid metabolites, their molecular diversity, redox activity, and non-overlapping mechanisms render them of interest for complementary drug development, particularly in oxidative stress–related disorders.

However, from a clinical perspective, the relevant evidence remains very limited, and there have been no reports of dedicated phase I/II clinical trials on purified phenolic or quinone derivatives. Current knowledge mainly comes from their presence in Tripterygium wilfordii glycoside preparations used in Chinese clinical practice.

Patient prognosis. In autoimmune diseases and kidney diseases, especially rheumatoid arthritis (RA), systemic lupus erythematosus (SLE), and nephrotic syndrome, patients treated with Tripterygium wilfordii preparations usually experience reduced joint swelling and pain, decreased proteinuria, and lower inflammatory markers (Chen et al., 2025). Although it cannot be directly attributed, these clinical outcomes may partially reflect the activity of phenolic and quinone metabolites.

Adverse reactions. The clinical use of these preparations is also associated with significant toxicity, including hepatotoxicity (elevated liver enzymes, liver dysfunction), gastrointestinal irritation (nausea, diarrhea, abdominal discomfort), reproductive toxicity (menstrual irregularities, reduced fertility), and hematological suppression (anemia, leukopenia). It is currently uncertain whether the phenolic and quinone derivatives specifically cause these toxicities, but their reactive quinone structures suggest potential risks of hepatotoxicity and cytotoxicity (Chen et al., 2025).

In summary, phenolic and quinone derivatives show promising pharmacological properties, but there is a lack of independent clinical trial data. Their impact on the efficacy and toxicity of Tripterygium wilfordii preparations highlights the need for systematic early - stage clinical trials to evaluate pharmacokinetics, safety, and therapeutic benefits.

#### 2.3.3 Pharmacological relevance and development potential

Although the alkaloids, phenolic metabolites, and quinone derivatives derived from *Tripterygium wilfordii* represent minor metabolites in terms of abundance and pharmacological intensity, they exhibit several distinctive features that support their continued investigation. These metabolites possess unique structural scaffolds not commonly found in the major diterpenoid or triterpenoid classes, and in some cases, demonstrate biological activities that may complement or extend the therapeutic scope of the plant’s traditional active metabolites.

Several alkaloids, including wilfordine and wilformine, have demonstrated immunomodulatory and antioxidant effects with markedly lower cytotoxicity than metabolites such as triptolide (Bresciani et al., 2023). This favorable toxicity profile may allow for prolonged administration or combination therapy, particularly in chronic autoimmune or inflammatory diseases. Phenolic and quinone derivatives, such as wilfordinol and triptonil, have shown the capacity to modulate oxidative stress and inflammatory signaling via suppression of NF-κB and MAPK activation (Wang and He, 2022). These effects may be relevant in conditions such as neuroinflammation, atherosclerosis, or metabolic dysfunction, where redox imbalance is a key pathogenic factor.

From a drug development perspective, the chemical diversity and pharmacophoric flexibility of these metabolites are notable. The presence of electrophilic quinone rings, nitrogen atoms, and aromatic hydroxyl groups allows for rational derivatization aimed at improving solubility, bioavailability, or target selectivity (Zhu et al., 2021). In particular, the relatively low molecular weight and favorable drug-like properties of alkaloids and phenolics make them attractive candidates for early-stage screening libraries. Nonetheless, these minor metabolites remain understudied, with sparse data on *in vivo* efficacy, ADME properties, and specific molecular targets. For instance, triptonil has shown chemical instability under physiological pH, which limits its translational potential without structural modification (Yijun et al., 2024). Moreover, the electrophilic nature of some quinones may result in nonspecific protein alkylation, posing toxicity risks if not adequately controlled (Chang et al., 2014).

In conclusion, while not as potent as the canonical terpenoid metabolites, these structurally diverse metabolites contribute to the broader pharmacological spectrum of *T. wilfordii*. Their distinct modes of action, lower toxicity, and unexploited chemical space provide a compelling rationale for further exploration as complementary agents or templates in natural product–based drug discovery (Table 1).

**TABLE 1:** Overview of major metabolites.

Chemical class	Compound	CAS no.	Mechanistic targets/Pathways	Potential applications	Clinical status	Source in *T. wilfordii*	References
Diterpenoids	Triptolide	38748-32-2	NF-κB, DNA pol II, PI3K/AKT	RA, cancer, SLE	Phase I (minnelide)	Root bark	Qiu et al. (1999)
	Triptonide	38647-11-9	Oxidative stress, apoptosis	Inflammation, tumor	Preclinical	Root bark	Song et al. (2023)
	Tripdiolide	39027-92-0	NF-κB inhibition, anti-inflammatory	Autoimmunity	Preclinical	Root bark	Xiang et al. (2020)
	Triptobenzene A	121848-75-7	Mitochondrial dysfunction	Inflammation	Preclinical	Root bark	Chen et al. (2025)
	Triptogelin	120350-38-1	NF-κB inhibition	Kidney disease	Preclinical	Root bark	Liu et al. (2021)
	Triptolidenol	118659-86-2	ROS-related pathways	Inflammation	Preclinical	Root bark	Lamers et al. (2022)
	Triptocassine	153360-87-1	Anti-fibrosis	Liver/kidney fibrosis	Preclinical	Root bark	Manautou (2003)
	Triptobifuran A/B	144787-09-3 / 144787-10-6	ROS modulation	Autoimmune disorders	Preclinical	Root bark	Li et al. (2023)
Triterpenoids	Celastrol	34157-83-0	HSP90, NF-κB, leptin	Obesity, cancer	Preclinical	Root bark	Shin et al. (2020)
	Pristimerin	1257-25-4	ROS, caspase	Cancer, inflammation	Preclinical	Root bark	Lee et al. (20216)
	Triptonoterpene	865296-41-7	NF-κB, MAPK	Inflammation	Preclinical	Root bark	Zhang X et al. (2021)
	Wilforlide A	106411-31-8	Smad2/3, TGF-β	Fibrosis	Preclinical	Cortex	Li J. J. et al (2019)
	Wilforlide B	142409-85-6	TGF-β	Fibrosis	Preclinical	Cortex	
	Wilfordic acid	133416-60-1	MMPs, ECM	Diabetic nephropathy	Preclinical	Root bark	
	Wilfoside K1N	131629-98-0	Glucose metabolism	Diabetic nephropathy	TG extract in use	Root cortex	
	Wilfoside C1N	131629-99-1	Fibrosis modulation	CKD, SLE	TG extract in use	Root cortex	
	Triptotriterpenic acid A	122297-93-2	TGF-β signaling	Fibrosis	Preclinical	Root bark	Zhou et al. (2025)
	Triptotriterpenic acid B	122297-94-3	ECM, fibronectin	Fibrosis	Preclinical	Root bark	Zhou et al. (2025)
Alkaloids	Wilformine	15301-93-6	ROS, calcium	Neuropathy	Not evaluated	Root	Cao et al. (2022)
	Tripterine	484-01-3	Calcium inhibition	Inflammation	Preclinical	Leaf	Fu et al. (2021)
	Triptonine	114418-34-7	Neural protection	Neurodegeneration	Preclinical	Root bark	Aivazian et al. (2017)
Phenolics/Quinones	Triptocassine B	163939-16-0	TGF-β, ROS	Fibrosis	Preclinical	Cortex	Chen et al. (2020)
	Triptonolide	147851-01-0	MAPK, oxidative stress	Inflammation	Preclinical	Leaf	He et al. (2022)
	Tripterygium glycoside I/II	N/A	TGF-β/Smad	DKD, SLE	In clinical TG extract	Cortex/leaf	Fellman et al. (2019)

## 3 Molecular mechanisms and therapeutic targets

The pharmacological versatility of Tripterygium wilfordii-derived metabolites stems from their capacity to interact with a diverse array of molecular targets involved in inflammation, immunity, oxidative stress, apoptosis, fibrosis, and metabolic regulation. These interactions form the basis for their therapeutic potential in a wide range of complex disorders, including autoimmune diseases, cancer, metabolic syndrome, and organ fibrosis. Among these metabolites, triptolide, celastrol, and specific triterpenoid alkaloids have been the most thoroughly characterized, highlighting both shared and unique mechanistic pathways.

### 3.1 Immunomodulation and oxidative stress

A hallmark of several metabolites, particularly triptolide and celastrol, is their potent ability to suppress inflammation via inhibition of nuclear factor-κB (NF-κB), Janus kinase/signal transducers and activators of transcription (JAK/STAT), and mitogen-activated protein kinase (MAPK) pathways (Panipinto et al., 2021). Triptolide downregulates pro-inflammatory cytokines such as TNF-α, IL-1β, IL-6, and IL-17 through inhibition of IκB kinase and blockade of NF-κB p65 nuclear translocation. It also reduces Th17 cell differentiation while promoting regulatory T cell (Treg) expansion (Fang et al., 2021), offering utility in autoimmune diseases like rheumatoid arthritis and systemic lupus erythematosus.

Celastrol exerts anti-inflammatory effects by targeting both canonical and non-canonical NF-κB pathways and interfering with MAPK signaling cascades including ERK, JNK, and p38. It also inhibits STAT3 activation, which plays a central role in immune cell proliferation and survival (Pa et al., 2023). Moreover, celastrol is capable of reducing macrophage infiltration and inflammatory cytokine production in models of colitis and asthma (Cong et al., 2022). Another important immune-related mechanism involves the suppression of inflammasome activation. Triptolide inhibits NLRP3 inflammasome assembly by preventing ASC oligomerization and caspase-1 activation, thereby reducing IL-1β and IL-18 secretion in models of lupus nephritis and diabetic kidney disease (Fu and Wu, 2023). This positions T. wilfordiimetabolites as attractive candidates for diseases characterized by innate immune overactivation.

Metabolites such as celastrol, wilformine, and wilfordinol modulate redox homeostasis through multiple mechanisms. Celastrol functions as a pro-electrophilic compound that modifies Keap1 cysteine residues, liberating Nrf2 and enhancing transcription of antioxidant response genes like HO-1, NQO1, and GCLC (Kurzawski et al., 2012). This Nrf2 pathway activation contributes to cytoprotection in cardiovascular, metabolic, and neurodegenerative disease models.Wilformine and other alkaloids have been shown to reduce intracellular ROS accumulation and maintain mitochondrial membrane integrity under oxidative stress conditions (Wang M. et al., 2020).The interplay between oxidative stress and inflammation also positions these metabolites for use in diseases involving mitochondrial dysfunction and oxidative damage, such as Alzheimer’s disease, Parkinson’s disease, and inflammatory bowel disease.

### 3.2 Apoptosis, cell cycle arrest, and DNA damage response

Apoptotic induction is a core anticancer mechanism for triptolide, which activates both intrinsic and extrinsic cell death pathways. Triptolide enhances expression of pro-apoptotic Bax, cleaved caspase-3, and cytochrome c release, while downregulating anti-apoptotic proteins such as Bcl-2, XIAP, and survivin (Zhang Y. et al., 2021). In addition, triptolide induces S-phase and G2/M cell cycle arrest by downregulating cyclins (e.g., Cyclin D1) and upregulating p21 and p27.

Crucially, triptolide targets the XPB subunit of the transcription factor IIH (TFIIH) complex, thereby disrupting transcription-coupled nucleotide excision repair (TC-NER) and RNA polymerase II function (Son and Scharer, 2020). This transcriptional shutdown sensitizes cancer cells to DNA-damaging agents such as cisplatin and ionizing radiation (Wang Y. et al., 2022). These mechanisms have been validated in various tumor models, including pancreatic, hepatocellular, breast, and hematologic malignancies.

Celastrol also demonstrates anti-proliferative activity by inducing ER stress and autophagy-mediated cell death, particularly in glioma and leukemia cells. It modulates unfolded protein response (UPR) signaling by upregulating CHOP and PERK and downregulating GRP78 (Treloar et al., 2002).

### 3.3 Metabolic regulation and anti-fibrotic activity

Recent studies have expanded the therapeutic scope of T. wilfordiimetabolites to include metabolic and fibrotic diseases. In high-fat diet and leptin-resistant obesity models, celastrol restores leptin sensitivity and promotes weight loss by modulating hypothalamic inflammation and mitochondrial thermogenesis (Wang Y. et al., 2022). It activates AMPK signaling and enhances expression of UCP1 in brown adipose tissue, indicating systemic metabolic benefits.

Triptolide and celastrol have both been shown to attenuate fibrogenic responses in models of renal, hepatic, and pulmonary fibrosis. Mechanistically, they suppress TGF-β1/Smad3 signaling, inhibit epithelial-mesenchymal transition (EMT), and reduce extracellular matrix deposition of fibronectin and type I/III collagen (Liu et al., 2023). In cardiac models, celastrol inhibits STAT3 phosphorylation and reduces myofibroblast activation, thereby reversing myocardial fibrosis (Gilham et al., 2023).

**FIGURE 1:**
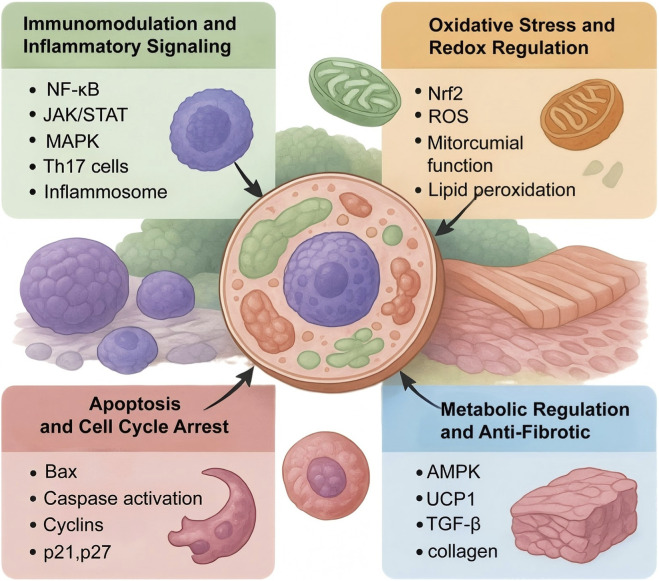
Mechanisms of action and signaling pathways of major bioactive metabolites from *Tripterygium wilfordii* **.** Key metabolites such as triptolide and celastrol exert therapeutic effects through multiple pathways, including inhibition of NF-κB, JAK/STAT, and MAPK signaling, activation of the Nrf2 antioxidant response, suppression of TGF-β/Smad-mediated fibrosis, and regulation of apoptosis, cell cycle, and energy metabolism. These multi-target interactions underpin their potential in treating autoimmune, inflammatory, metabolic, and fibrotic diseases.

These findings highlight a unique aspect of T. wilfordii metabolites—their ability to simultaneously regulate immune, metabolic, and fibrotic axes, making them attractive for complex syndromes such as diabetic kidney disease, NASH, and systemic sclerosis (Figure 1).

## 4 Clinical applications and translational progress

The transition of Tripterygium wilfordii-derived metabolites from bench to bedside has been propelled by their broad pharmacological actions and accumulating preclinical evidence. However, clinical translation has encountered significant barriers, particularly regarding safety, pharmacokinetics, and formulation strategies. Despite these challenges, a growing number of clinical and translational efforts are exploring both traditional and modern applications of key metabolites such as triptolide and celastrol, as well as their derivatives.

The traditional and modern applications of key metabolites such as tripterine and celastrol and their derivatives.

Tripterygium wilfordii and its active metabolites have been studied in various diseases, but their clinical applications vary greatly depending on the indications.

- Cancer: Minnelide, a water - soluble prodrug of triptolide, has entered phase I/II clinical trials for pancreatic cancer and acute myeloid leukemia (AML). Partial remission and disease stabilization have been observed in some patients, but myelosuppression and gastrointestinal intolerance are dose - limiting toxicities. Celastrol has shown efficacy in pre - clinical studies of breast cancer and prostate cancer, but no human oncology trials have been conducted yet (Hussain et al., 2025).
- Autoimmune diseases: Tripterygium glycosides tablets are widely used in China to treat rheumatoid arthritis (RA) and systemic lupus erythematosus (SLE). Clinical efficacy includes reducing joint pain, lowering disease activity scores, and improving proteinuria, and the effects are generally comparable to those of standard immunosuppressants. However, long - term use is limited by hepatotoxicity, reproductive toxicity, and hematological suppression (Chen et al., 2025).
- Metabolic disorders: Celastrol has emerged as a potential therapy for obesity and type 2 diabetes mellitus (T2DM). In phase I and phase II pilot trials, celastrol can reduce appetite, lead to moderate weight loss, and improve insulin sensitivity. Gastrointestinal side effects, hepatotoxicity, and fatigue are the most common adverse events (Zheng et al., 2024).
- Fibrotic diseases: Pre - clinical studies have shown that triptolide and celastrol can alleviate renal and pulmonary fibrosis by regulating the TGF - β/Smad and NF - κB signaling pathways. Although the prospects are promising, there is currently a lack of clinical data, highlighting the gap in translational applications in this field (Wen et al., 2024).

In summary, although Tripterygium - derived metabolites show therapeutic potential in multiple disease areas, their clinical applications are most mature in cancer, autoimmune diseases, and metabolic disorders, while they are still in the pre - clinical stage for fibrotic and neurodegenerative diseases. Safety issues, especially hepatotoxicity and reproductive toxicity, remain the main obstacles to broader clinical translation.

To fill the gap in translational evidence, we included additional *in vivo* studies on less - studied metabolites. For example, triptriolide has shown immunosuppressive effects in mouse models of autoimmune encephalomyelitis and collagen - induced arthritis (Wen et al., 2024). It reduced the levels of inflammatory cytokines and alleviated the disease severity, despite the lack of formal clinical trials. Similarly, maytansine has demonstrated anti - tumor efficacy in xenograft models of breast and prostate cancer. It induces apoptosis and inhibits tumor growth through the PI3K/Akt and NF - κB pathways. Triptolide has been tested in rodent models of nephritis and arthritis. It improved renal function indicators and reduced joint swelling, which supports its potential immunomodulatory effects (Wen et al., 2024). However, despite these encouraging pre - clinical findings, early human data remain scarce. Rigorous phase I safety evaluations are urgently needed to determine pharmacokinetics, therapeutic index, and dose - limiting toxicities.

### 4.1 Clinical applications in autoimmune and inflammatory disorders

Triptolide and its parent extract (Tripterygium glycosides) have long been employed in traditional Chinese medicine to manage autoimmune diseases, particularly rheumatoid arthritis (RA), systemic lupus erythematosus (SLE), and nephrotic syndrome. Modern clinical trials have substantiated these effects, demonstrating significant reductions in proteinuria, inflammatory markers (e.g., ESR, CRP), and disease activity scores in RA and SLE patients receiving TG-based therapy as adjuncts to conventional immunosuppressants (Mooventhan et al., 2023).

One multicenter randomized trial showed that a TG-containing regimen provided similar therapeutic efficacy to methotrexate in RA patients, with added benefits in joint pain reduction and erythrocyte sedimentation rate (ESR) decline (Ishiguro et al., 2013). In lupus nephritis, triptolide reduced anti-dsDNA titers and renal immune complex deposition, indicating immunomodulatory and renal-protective properties (Ma et al., 2023).

Nonetheless, long-term clinical use is limited by hepatic and reproductive toxicity, prompting the need for lower-dose formulations, therapeutic monitoring, and combination approaches to minimize adverse effects.

### 4.2 Expanding therapeutic Frontiers: oncology, metabolism, and fibrosis

The potent anti-tumor activity of triptolide has catalyzed the development of Minnelide, a water-soluble triptolide prodrug that has advanced into phase I/II clinical trials for pancreatic cancer, osteosarcoma, and refractory solid tumors (Tsimberidou et al., 2022). Early clinical data suggest favorable bioavailability and partial tumor responses at tolerable doses, although dose-limiting toxicities (notably gastrointestinal and hematologic) remain a concern.Celastrol has shown strong preclinical efficacy across multiple malignancies, including glioma, prostate cancer, and leukemia. However, no celastrol-based therapies have yet entered clinical trials, largely due to its narrow therapeutic window and poor solubility. Recent efforts in nanocarrier systems and prodrug synthesis are under investigation to improve delivery and tumor targeting (Fattahi et al., 2020).

Animal studies have shown that celastrol improves leptin sensitivity, reduces weight gain, and enhances glucose tolerance in diet-induced obesity models. These findings have led to pilot clinical trials assessing celastrol’s metabolic effects in human obesity and type 2 diabetes, although results remain unpublished (Liu et al., 2015). Its capacity to regulate hypothalamic inflammation and mitochondrial bioenergetics suggests broader utility in metabolic syndrome.Both triptolide and celastrol demonstrate anti-fibrotic activity in models of renal, hepatic, and cardiac fibrosis. Though not yet tested clinically for these indications, their inhibition of TGF-β/Smad and STAT3 signaling presents a rational foundation for trials in chronic kidney disease (CKD), non-alcoholic steatohepatitis (NASH), and idiopathic pulmonary fibrosis (IPF) (Eurich et al., 2012).

### 4.3 Formulation innovation, safety challenges, and regulatory prospects

The multi-targeted pharmacological nature of Tripterygium-derived metabolites offers both opportunities and challenges for clinical translation. The polypharmacology of T. wilfordiimetabolites, while contributing to efficacy in multifactorial diseases, also presents translational challenges. Issues such as poor solubility, organ-specific toxicity (especially hepatotoxicity and nephrotoxicity), and narrow therapeutic windows necessitate careful formulation and dosing strategies (Qian et al., 2022). Structure-based derivatization is also underway to preserve bioactivity while mitigating toxicity, particularly by modifying reactive electrophilic sites or enhancing target specificity. Moreover, combination therapies that utilize metabolites at sub-toxic doses alongside conventional drugs (e.g., immunosuppressants, chemotherapeutics, anti-fibrotic agents) are under investigation as a means of maximizing therapeutic benefit while minimizing adverse effects. In summary, the multitargeted molecular actions of T. wilfordii metabolites—spanning immune modulation, redox regulation, apoptosis, and fibrotic suppression—provide a compelling framework for their continued development. Rational design, mechanistic elucidation, and translational optimization will be key to unlocking their full clinical potential.

To overcome solubility and toxicity challenges, several delivery platforms are under development, including liposomes, polymeric nanoparticles, and cyclodextrin complexes. These approaches aim to enhance bioavailability, reduce organ-specific toxicity, and prolong circulation time (Pandey et al., 2021). Structural analogs such as Minnelide, and celastrol-derived hybrids with altered redox centers or reduced Michael acceptor activity, represent another major translational strategy. *In vitro* and *in vivo* screens have yielded promising leads with preserved efficacy and improved safety profiles (Zeng et al., 2023). Additionally, combination therapies leveraging synergistic pathways—e.g., triptolide plus cisplatin in cancer or celastrol plus metformin in metabolic disease—offer a rational path toward enhanced therapeutic indices.

**FIGURE 2:**
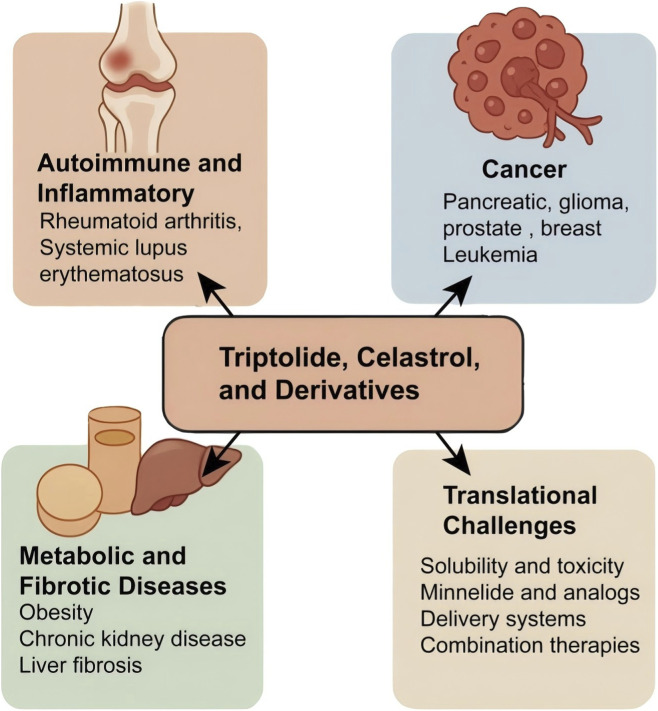
Therapeutic applications and translational challenges of *Tripterygium wilfordii*-derived metabolites. Metabolites including triptolide and celastrol have shown efficacy in rheumatoid arthritis, systemic lupus erythematosus, pancreatic and breast cancer, obesity, and kidney fibrosis. However, clinical translation remains limited by poor solubility, systemic toxicity, and low bioavailability. Current strategies such as prodrug development, nanocarrier delivery, targeted modifications, and combination therapies aim to overcome these barriers.

Despite potent bioactivity, T. wilfordiimetabolites are associated with hepatotoxicity, nephrotoxicity, and reproductive suppression in animal and human studies. As such, future clinical use will require comprehensive toxicological evaluation, biomarker-guided dosing, and rigorous post-marketing surveillance. Regulatory approval for derivatives like Minnelide may pave the way for next-generation TG-based drugs, contingent upon demonstration of safety margins and disease specificity.In summary, Tripterygium-derived metabolites possess substantial translational value across autoimmune, oncologic, and metabolic domains. Ongoing innovation in formulation, derivatization, and combinatorial strategies will be pivotal to unlock their full clinical potential (Figure 2).

### 4.4 Toxicity

Toxicity is the major limiting factor for the clinical translation of Tripterygium wilfordii and its active metabolites. The most clinically relevant adverse reactions include hepatotoxicity, reproductive toxicity, and myelosuppression, each of which has a direct impact on the treatment management of patients.

- Hepatotoxicity: The clinical use of tripterygium glycosides is often associated with elevated liver enzymes and hepatocyte damage, which can lead to liver dysfunction in severe cases. This toxicity is dose - dependent and may be caused by the active metabolites of diterpenoids and triterpenoids, which damage mitochondria and activate oxidative stress pathways. Clinically, this requires regular monitoring of liver function and often results in the interruption of long - term treatment (Zhou et al., 2025).
- Reproductive toxicity: Both the male and female reproductive systems are vulnerable. Women may experience menstrual irregularities or amenorrhea, while men may have a decrease in sperm count and motility. Although triptolide has been studied as a potential non - hormonal male contraceptive, these reproductive effects have become a major obstacle to the broader application of Tripterygium wilfordii preparations in chronic diseases, especially for patients of child - bearing age (Xu et al., 2019).
- Myelosuppression: Hematological toxicity is manifested as leukopenia, anemia, and thrombocytopenia, which increase the risk of infection, fatigue, and bleeding. This toxicity is thought to be related to the inhibition of the proliferation of hematopoietic progenitor cells, which is consistent with the potent immunosuppressive activity of Tripterygium wilfordii. Clinically, myelosuppression limits the safe duration and cumulative dose of treatment, requires hematological monitoring, and in some cases, supportive interventions (Xu et al., 2019).

In summary, hepatotoxicity, reproductive toxicity, and myelosuppression are interrelated with the immunosuppressive and cytotoxic properties of Tripterygium wilfordii. These toxicities greatly limit its therapeutic window. Therefore, careful dose adjustment, safety monitoring, and exploration of drug delivery systems or derivatives that can reduce systemic toxicity while retaining efficacy are needed.

### 4.5 Nanodelivery systems and prodrug design

Due to poor water solubility, rapid in - vivo clearance, and a narrow therapeutic window, both triptolide and celastrol have been extensively studied through nanodelivery systems and prodrug strategies to improve their pharmacological properties.

Triptolide has problems of extremely high toxicity and poor solubility. Several prodrug methods have been developed so far, and the most notable one is Minnelide, a water - soluble phosphate prodrug that is currently undergoing Phase I/II trials for pancreatic cancer and acute myeloid leukemia. Minnelide improves water solubility and reduces acute systemic toxicity, but myelosuppression and gastrointestinal intolerance remain the main adverse events (Yuan et al., 2024).

In terms of nanocarriers, liposomes and polymer nanoparticles (such as polyethylene glycol - polylactic acid [PEG - PLA], poly(lactic - co - glycolic acid) [PLGA]) have shown promise in prolonging the circulation time and enhancing tumor accumulation. Liposomal triptolide improves the in - vivo distribution of the drug, but its drug - loading capacity is limited; while polymer nanoparticles improve stability, they may cause immunogenicity problems (Yuan et al., 2024).

Celastrol is limited by poor solubility and gastrointestinal/liver toxicity. Nanopreparations such as liposomes, solid lipid nanoparticles (SLNs), and polymer micelles improve its oral bioavailability and reduce gastrointestinal irritation. Liposomes can achieve controlled drug release, but they may be unstable during storage; while solid lipid nanoparticles and micelles improve dispersibility, they have limitations in large - scale production (Zhang et al., 2023).

Prodrug strategies, such as esterification and pegylation, improve the solubility of celastrol and reduce off - target toxicity. However, the variability of metabolic activation and potential loss of activity remain unsolved problems.

Summary: Triptolide benefits most from the development of prodrugs (Minnelide) and tumor - targeting nanoparticles, while celastrol has made greater progress in nanopreparations (liposomes, solid lipid nanoparticles, micelles) for the treatment of metabolic diseases. Each method improves solubility and safety, but also faces obstacles in scalability, long - term stability, and toxicity reduction.

## 5 Conclusion and future perspectives

Major bioactive metabolites derived from *Tripterygium wilfordii*—notably triptolide, celastrol, and a range of diterpenoids and triterpenoids—have demonstrated broad-spectrum biological activities including immunomodulation, anti-inflammation, anti-fibrosis, metabolic regulation, and tumor suppression. Mechanistically, these effects are mediated through modulation of critical signaling pathways such as NF-κB, JAK/STAT, MAPK, and TGF-β/Smad, rendering these metabolites attractive candidates for intervention in complex and refractory diseases (Park et al., 2015) (Figure 4).

Nevertheless, clinical translation is currently constrained by poor aqueous solubility, low bioavailability, narrow therapeutic windows, and systemic toxicity. In response, emerging strategies such as prodrug development (e.g., Minnelide), nanocarrier-based delivery systems, and structural analog synthesis have been proposed to overcome these pharmacokinetic and safety hurdles (Wright et al., 2022). Future research should focus on rational structure–activity relationship (SAR) refinement through synthetic modification and computational modeling, aiming to develop analogs with enhanced efficacy and reduced toxicity (Koval et al., 2022). In parallel, advancements in targeted delivery technologies—particularly nanocarriers and ligand-conjugated platforms—may enhance tissue specificity and minimize systemic exposure (Warren and Oselin, 2011).

Expanding therapeutic indications via systems pharmacology and network-based analysis could unlock new clinical applications, particularly in metabolic disorders, immune-oncology, and fibrotic diseases. Clinical validation through standardized, biomarker-guided trials remains pivotal to secure global regulatory approval (Yatham, 2023). Moreover, integration of GMP-compliant manufacturing processes and quantitative quality control will be essential to ensure consistency and safety in large-scale use.

**FIGURE 3:**
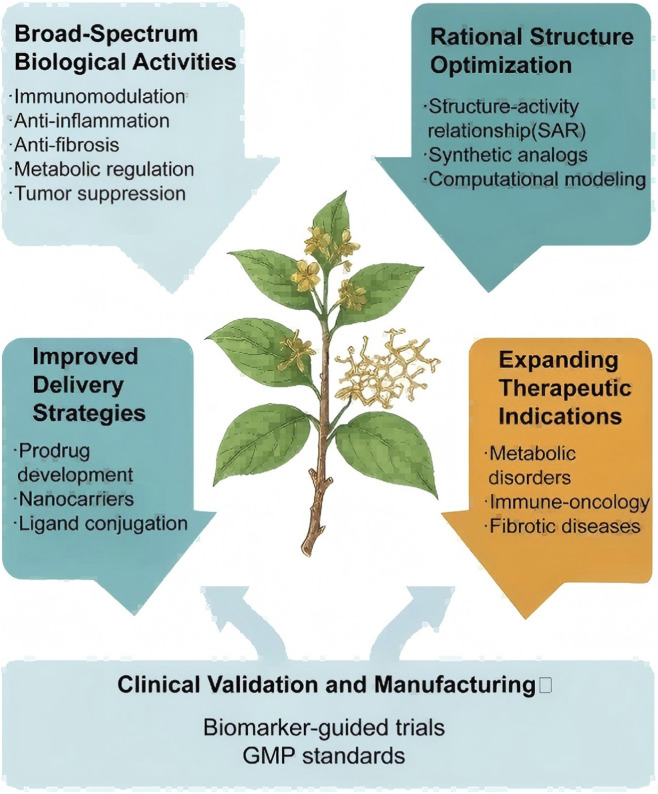
Future directions in the development of *Tripterygium wilfordii* compound-based therapeutics. Research is advancing toward structural optimization, efficient delivery systems, and target-specific interventions. Structure–activity relationship studies, synthetic modification, and precision drug delivery approaches are being explored to enhance efficacy and safety. Integration of biomarker-guided therapy, multi-omics profiling, and GMP-compliant production will be essential for clinical translation across chronic inflammatory, neoplastic, and metabolic disorders.

In summary, *T. wilfordii*-derived metabolites represent a valuable class of multi-target agents bridging traditional herbal pharmacology and modern biomedicine. Continued interdisciplinary efforts integrating chemical optimization, mechanistic exploration, translational medicine, and regulatory science will be key to realizing their full clinical and therapeutic potential (Figure 3).

**FIGURE 4:**
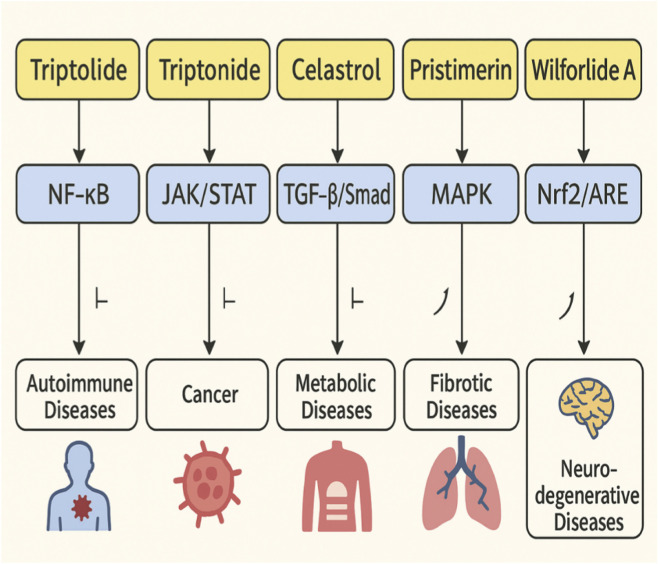
This diagram illustrates the therapeutic potential of several natural metabolites—Triptolide, Triptonide, Celastrol, Pristimertin, and Wilforlide A. It shows their interaction with key signaling pathways (NF-κB, JAK/STAT, TGF-β/Smad, MAPK, Nrf2/ARE), which are implicated in major diseases including autoimmune disorders, cancer, metabolic and fibrotic diseases, and neurodegenerative conditions. The chart summarizes a research framework linking these bioactive molecules to disease modulation through cellular pathways.

**FIGURE 5:**
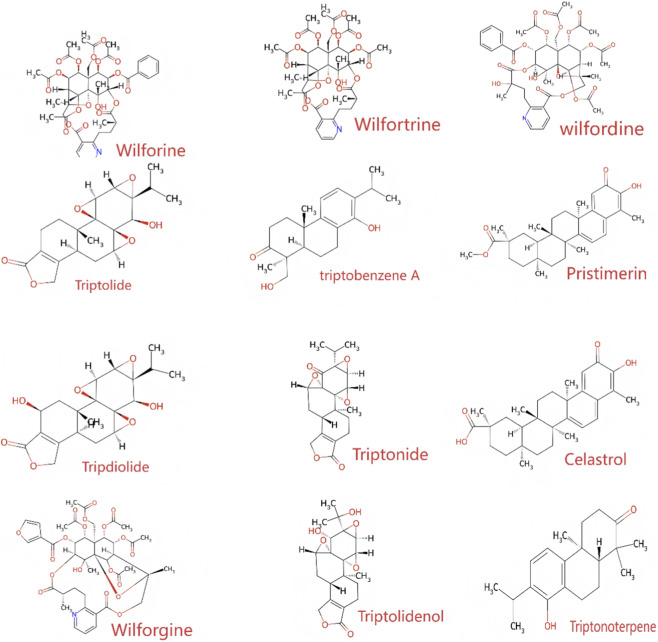
Chemical structures of representative bioactive metabolites isolated from Tripterygium wilfordii, including alkaloids, diterpenoids, and triterpenoids.

## 6 Limitations and critical assessment of current evidence

While the reviewed studies demonstrate promising pharmacological activities of *T. wilfordii* metabolites, several methodological limitations must be acknowledged. First, most *in vitro* studies utilize metabolite concentrations (e.g., triptolide at 50–200 nM) that are not achievable *in vivo* due to poor bioavailability, raising questions about the physiological relevance of many mechanistic claims. Second, the majority of *in vivo* animal studies lack blinded outcome assessment, randomization, or positive control groups, increasing the risk of bias (Huang et al., 2020). Third, clinical data are extremely limited: only Minnelide and celastrol have entered early-phase trials, and these studies suffer from small sample sizes, open-label designs, and high dropout rates due to toxicity. Fourth, systematic comparisons across different metabolites or across different disease models are largely absent. Future research must prioritize rigorous, pre-registered preclinical studies with clinically relevant dosing, as well as well-powered, double-blind, placebo-controlled clinical trials to establish true efficacy and safety profiles (Song et al., 2020).

Future research priorities include: (1) standardized *in vivo* models with clinically translatable dosing; (2) rigorous Phase I/II trials for promising metabolites like triptonide and tripdiolide; (3) development of targeted delivery systems to reduce off-target toxicity; (4) biomarker-guided patient selection to improve therapeutic index.
